# A Text-Book of Pathology

**Published:** 1901-03

**Authors:** 


A TEXT-BOOK OF PATHOLOGY. By ALFRED STENGEL, M. D., Professor of Clinical Medicine in the University of Pennsylvania; Physician to the Philadelphia Hospital; Physician to the Children's Hospital, Philadelphia, etc. Octavo, pp. 873, with 372 illustrations. Third edition, revised. Philadelphia and London: W. B. Saunders & Co. 1900. [Price, cloth, $5.00 net; half morocco, $6.00 net.]

The first edition of Stengel's pathology was reviewed in the July number, 1899, of the JOURNAL, and its many excellent points of superiority there noted. The third edition being demanded in the short space of two years, is but an indication that it has found great favor among the medical colleges and the profession at large. The present edition has been somewhat enlarged, and revised with the intention of bringing the subject-matter up-to-date and of amplifying the sections on pathologic physiology. The general arrangement of the subject-matter remain practically the same as in the preceding editions. The chapter on the pathology of the nervous system has been thoroughly revised and for this purpose the author has obtained the valuable services of Dr. Joseph Sailer, of Philadelphia, who has succeeded admirably in the difficult field of pathology.

The binding and press-work are similar to the preceding edition.

